# Outcome for Children with Metastatic Solid Tumors over the Last Four Decades

**DOI:** 10.1371/journal.pone.0100396

**Published:** 2014-07-08

**Authors:** Stephanie M. Perkins, Eric T. Shinohara, Todd DeWees, Haydar Frangoul

**Affiliations:** 1 Department of Radiation Oncology, Washington University School of Medicine, Saint Louis, Missouri, United States of America; 2 Department of Radiation Oncology, Vanderbilt University School of Medicine, Nashville, Tennessee, United States of America; 3 Department of Pediatrics, Vanderbilt University School of Medicine, Nashville, Tennessee, United States of America; Johns Hopkins University, United States of America

## Abstract

**Background:**

Outcomes for pediatric solid tumors have significantly improved over the last 30 years. However, much of this improvement is due to improved outcome for patients with localized disease. Here we evaluate overall survival (OS) for pediatric patients with metastatic disease over the last 40 years.

**Procedure:**

The United States Surveillance, Epidemiology, and End Results (SEER) database was used to conduct this study. Patients diagnosed between 0 and 18 years of age with metastatic Ewings sarcoma, neuroblastoma, osteosarcoma, rhabdomyosarcoma or Wilms tumor were included in the analysis.

**Results:**

3,009 patients diagnosed between 1973–2010 met inclusion criteria for analysis. OS at 10 years for patients diagnosed between 1973–1979, 1980–1989, 1990–1999 and 2000–2010 was 28.3%, 37.2%, 44.7% and 49.3%, respectively (p<0.001). For patients diagnosed between 2000–2010, 10-year OS for patients with Ewing sarcoma, neuroblastoma, osteosarcoma, rhabdomyosarcoma and Wilms tumor was 30.6%, 54.4%, 29.3%, 27.5%, and 76.6%, respectively, as compared to 13.8%, 25.1%, 13.6%, 17.9% and 57.1%, respectively, for patients diagnosed between 1973–1979. OS for neuroblastoma significantly increased with each decade. For patients with osteosarcoma and Ewing sarcoma, there was no improvement in OS over the last two decades. There was no improvement in outcome for patients with rhabdomyosarcoma or Wilms tumor over the last 30 years.

**Conclusions:**

OS for pediatric patients with metastatic solid tumors has significantly improved since the 1970s. However, outcome has changed little for some malignancies in the last 20–30 years. These data underscore the importance of continued collaboration and studies to improve outcome for these patients.

## Introduction

With advances in medical therapy, survival for pediatric solid tumors has significantly improved over the past 30 years. This improvement has been achieved through improvements in efficacy and reductions in toxicity of multi-modal therapy that includes chemotherapy, surgery and/or radiation therapy. While patients with localized disease have experienced the largest improvement in outcome, improvement in survival for patients with metastatic disease has been more limited [1]–[6].

The approach to treating pediatric patients with metastatic disease is unique from that of adults in that treatment is often approached with curative intent with the use of intensive therapy. Through pediatric cooperative groups in both the United States and abroad, numerous clinical trials including phase III randomized trials, have been completed or are currently being conducted. These studies not only allow for the inclusion of patients with metastatic disease but, in many cases, are also specifically designed for this subset of patients [7]–[12].

There has clearly been an effort to improve outcome for pediatric patients with metastatic disease. Using National Cancer Institute's Surveillance, Epidemiology, and End Results (SEER) registry, we evaluated outcome for patients less than 19 years of age diagnosed with metastatic Ewing sarcoma, neuroblastoma, osteosarcoma, rhabdomyosarcoma and Wilms tumor between 1973–2010.

## Materials and Methods

The United States Surveillance, Epidemiology, and End Results (SEER) database was used to conduct this study [13]. This research was determined to be exempt from IRB oversight by the IRB at Washington University School of Medicine. SEER*Stat version 8.1.2 (Surveillance Research Program, National Cancer Institute, Bethesda, MD, USA) was used to compile data from the SEER public-use database. Patients diagnosed between 1973–2010 provided by 18 registries were identified. Patients between 0 and 18 years of age diagnosed with metastatic disease from the following malignancies were included in the study: Ewing sarcoma, neuroblastoma, osteosarcoma, rhabdomyosarcoma and Wilms tumor. The following International Classification for Childhood Cancer site recode extended ICD-0-3 histology codes were included: 8900–8902, 8910, 8912, 8920, 8921, 8960, 9180–9183, 9186, 9192, 9193, 9260 and 9500. Patients with a prior diagnosis of cancer were excluded from analysis. Patient demographic information including age, race, gender, year of diagnosis, follow-up time, vital status at last follow-up and cause of death were collected. Overall survival (OS) was evaluated using the Kaplan-Meier method. Hazard ratios (HRs) for risk of mortality were calculated comparing patients by decade of diagnosis using the Cox proportional hazards model. Statistical analysis was performed using SAS version 9.2 (Cary, NC).

## Results

A total of 10,938 pediatric patients with Ewing sarcoma, neuroblastoma, osteosarcoma, rhabdomyosarcoma, and Wilms tumor were identified. Of these patients, 3,009 patients had metastatic disease at diagnosis and met inclusion criteria for analysis. Patient demographic information is presented in Table 1. Overall, 27.5% of patients were found to have metastatic disease at diagnosis. The percentage of patients presenting with metastatic disease did not significantly change over time ranging from 26% in the 1970s to 27% in the 2000s. The rate of metastatic disease was 22.3% for Ewing sarcoma, 49.1% for neuroblastoma, 22.5% for rhabdomyosarcoma, 13.1% for osteosarcoma and 20.4% for Wilms tumor. However, the number of children with metastatic disease included in the SEER registry increased with each decade. This is a reflection of the increasing number of SEER registry sites. In the 1970′s there were 9 SEER registry sites and by the end of this study period, there were 18 SEER registry sites.

**Table 1:** Patient Information.

	All Patients *N*(%)	Ewing Sarcoma	Neuroblastoma	Osteosarcoma	habdomyosarcoma	Wilms Tumor
Patients *N* (%)	3009	289 (9.6)	1478 (49.1)	266 (8.8)	441 (14.7)	535 (17.8)
Mean Age in Years at Diagnosis (range)	5.6 (0–18)	12.7 (0–18)	2.4 (0–18)	13.3 (2–18)	9.0 (0–18)	4.1 (0–17)
**Gender**						
Male	1681 (55.9)	171 (59.2)	839 (56.8)	169 (63.5)	257 (58.3)	245 (45.8)
Female	1328 (44.1)	118 (40.8)	639 (43.2)	97 (36.5)	184 (41.7)	290 (54.2)
**Race**						
White	2359 (78.4)	263 (91.0)	1137 (76.9)	199 (74.8)	335 (76.0)	425 (79.4)
Black	297 (13.2)	6 (2.1)	186 (12.6)	43 (16.2)	76 (17.2)	86 (16.1)
Other	234 (7.8)	20 (6.9)	140 (9.5)	23 (8.6)	29 (6.6)	22 (4.1)
Unknown	19 (0.6)	0	15 (1.0)	1 (0.4)	1 (0.2)	2 (0.4)
**Year of Diagnosis**						
1973–1979	248 (8.2)	32 (11.1)	117 (7.9)	22 (8.3)	28 (6.3)	49 (9.2)
1980–1989	458 (15.2)	47 (16.3)	215 (14.5)	39 (14.7)	64 (14.5)	93 (17.4)
1990–1999	653 (21.7)	61 (21.1)	319 (21.6)	71 (26.7)	86 (19.5)	116 (21.7)
2000–2010	1650 (54.8)	149 (51.6)	827 (56.0)	134 (50.4)	263 (59.6)	277 (51.8)

Abbreviations: *N* = number.

**Figure 1:**
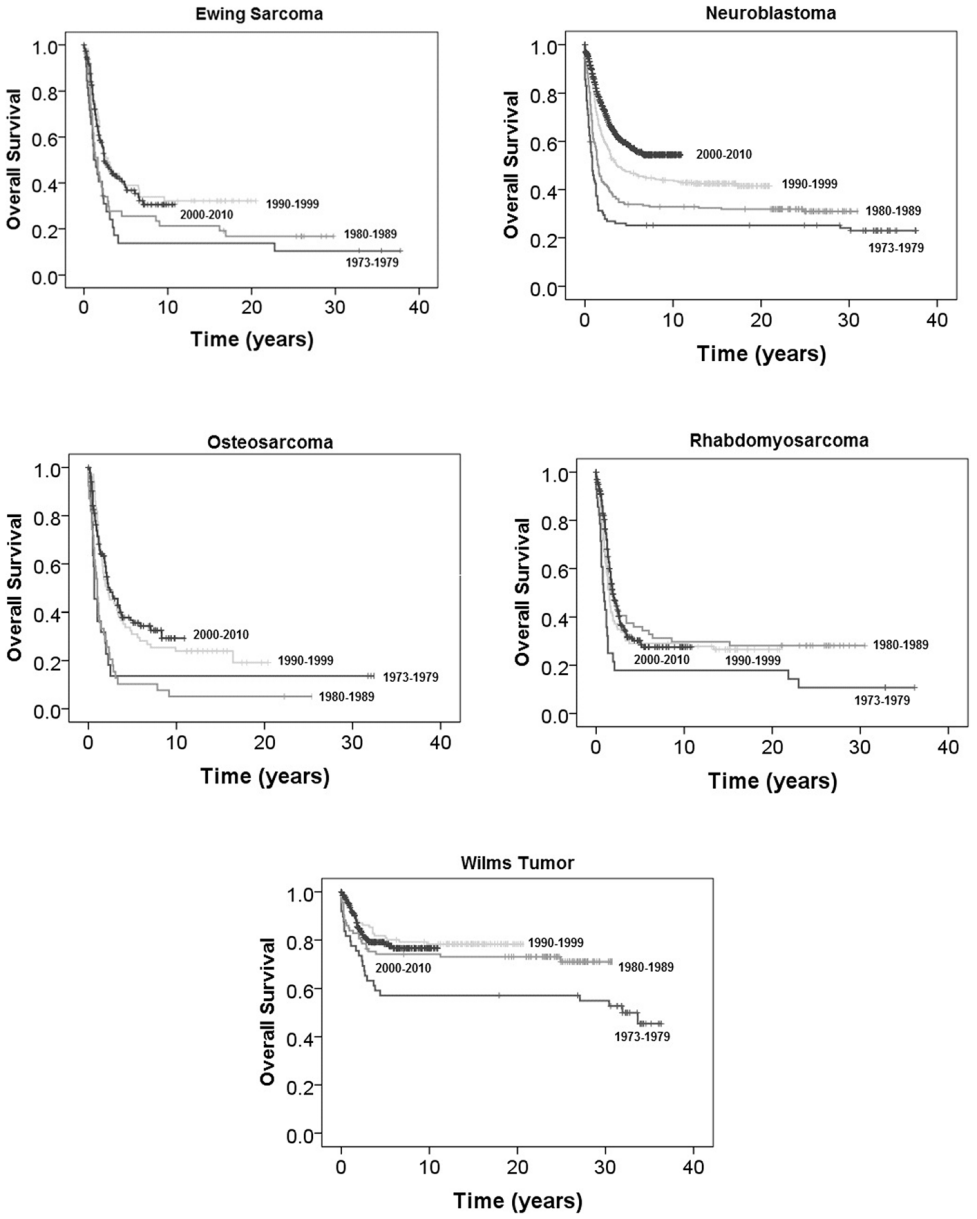
Overall survival based on tumor type.

Median follow-up of all patients was 2.3 years (range 0–37.8 years). OS over time for each tumor type is presented in Figure 1. HRs for risk of mortality OS for each tumor type over time are presented in Table 2. For patients with Ewing sarcoma, 10-year OS for patients diagnosed between 2000–2010 was 30.6% as compared to 13.8% for patients diagnosed between 1973–1979. Patients diagnosed between 1980–1989 experienced worse OS compared to patients diagnosed between 2000–2010 (HR = 1.48, 95% CI 1.01–2.17); however, there was no significant difference in OS for patients diagnosed in the 1990′s versus 2000–2010 (HR = 0.94, 95% CI 0.65–1.37).

**Table 2:** Hazard Ratio of overall survival for each tumor type based on decade of diagnosis.

	10-year Overall Survival (%)	Hazard Ratio of Overall Survival (95% CI)	*P* value
**Ewing Sarcoma**			
2000–2010	30.6	1.0	
1990–1999	32.2	0.94 (0.65–1.37)	0.75
1980–1989	21.3	1.48 (1.01–2.17)	0.046
1973–1979	13.8	1.84 (1.20–2.83)	0.005
**Neuroblastoma**			
*All Patients*			
2000–2010	54.4	1.0	
1990–1999	43.5	1.45 (1.20–1.74)	<0.0001
1980–1989	32.9	2.15 (1.76–2.63)	<0.0001
1973–1979	25.1	3.08 (2.43–3.92)	<0.0001
*<2 years old*			
2000–2010	71.5	1.0	
1990–1999	59.1	1.50 (1.10–2.05)	0.010
1980–1989	52.7	2.00 (1.44–2.79)	<0.0001
1973–1979	42.9	2.76 (1.89–4.01)	<0.0001
*≥2 years old*			
2000–2010	38.9	1.0	
1990–1999	26.8	1.41 (1.20–1.91)	0.0005
1980–1989	10.9	3.00 (2.34–3.86)	<0.0001
1973–1979	2.0	6.5 (4.73–9.00)	<0.0001
**Osteosarcoma**			
2000–2010	29.3	1.0	
1990–1999	23.9	1.11 (0.78–1.56)	0.57
1980–1989	5.1	2.31 (1.56–3.42)	<0.0001
1973–1979	13.6	2.14 (1.29–3.55)	0.003
**Rhabdomyosarcoma**			
2000–2010	27.5	1.0	
1990–1999	29.1	1.15 (0.86–1.54)	0.36
1980–1989	29.7	1.00 (0.72–1.40)	0.99
1973–1979	17.9	2.01 (1.31–3.08)	0.001
**Wilms Tumor**			
2000–2010	76.6	1.0	
1990–1999	78.3	0.91 (0.56–1.46)	0.91
1980–1989	74.2	1.22 (0.76–1.97)	0.40
1973–1979	57.1	2.11 (1.28–3.50)	0.003

Abbreviation: CI, Confidence Interval; NR, not yet reached.

For patients with neuroblastoma, OS significantly increased during each decade (Figure 1). When compared to patients diagnosed between 2000–2010, HRs for risk of mortality were significantly worse for each previous decade (Table 2). OS at 10 years for patients diagnosed between 1973–1979, 1980–1989, 1990–1999 and 2000–2010 were 25.1%, 32.9%, 43.5% and 54.4%, respectively (p<0.001). For neuroblastoma patients greater than or equal to 2 years old at diagnosis there is also steady significant improvement in outcome over time. In the 1970s and 1980s, 10-year OS for these older children was 2.0% and 10.9%, respectively. By the 1990s, 10-year OS increased to 26.8% and for patients diagnosed after 1999 10-year OS was 38.9%. Patients diagnosed prior the age of two years diagnosed between 1973–1979, 1980–1989, 1990–1999 and 2000–2010 experienced 10-year overall-survival of 42.9%, 52.7%, 59.1%, and 71.5%, respectively (p<0.001). For patients two years of age or older, OS between 1973–1979, 1980–1989, 1990–1999 and 2000–2010 was 2.0%,10.9%, 26.8%, and 38.9%, respectively (p<0.001). HRs for risk of mortality for neuroblastoma patients stratified by age less than 2 years of age are presented in Table 2.

OS at 10 years for osteosarcoma patients with metastatic disease diagnosed between 1973–1979, 1980–1989, 1990–1999 and 2000–2010 were 13.6%, 5.1%, 23.9% and 29.3%, respectively. Patients diagnosed between 2000–2010 experience improved survival over patients diagnosed in the 1970s and 1980s; however, there has been no significant improvement in outcome compared to patients treated in the 1990s.

For patients with metastatic rhabdomyosarcoma, 10-year OS for the past 30 years was nearly identical. OS at 10 years for patients diagnosed between 1980–1989, 1990–1999 and 2000–2010 was 29.7%, 29.1% and 27.5%, respectively. However, patients diagnosed between 2000–2010 did experience a significant increase in survival as compared to patients diagnosed between 1973–1979 (HR = 2.01, 95% CI 1.31–3.08). For patients with embryonal rhabdomyosarcoma (n = 192), OS at 10 years for patients diagnosed between 1973–1979, 1980–1989, 1990–1999, 2000–2010 was 27.8%, 37.2%, 35.0% and 40.5%, respectively. There were few patients with known alveolar histology diagnosed prior to 2000 (n = 46). For patients diagnosed between 2000–2010 with alveolar histology, 10-year OS was 19.5%.

Wilms tumor patients with metastatic disease diagnosed between 1973–1979, 1980–1989, 1990–1999 and 2000–2010 experienced 10-year OS of 57.1%, 74.2%, 78.3%, and 76.6%, respectively. Patients diagnosed between 1973–1979 experienced an increased risk of mortality compared to patients diagnosed between 2000–2010 (HR = 2.11, 95% CI 1.28–3.50). However, there has been no significant increase in survival since that time.

## Discussion

Our study shows that despite improvements in survival in children with metastatic diseases over the past four decades the outcome in some disease continues to be poor. This is especially true for patients with bone and soft tissue sarcoma. Rhabdomyosarcoma is the most common soft tissue sarcoma and a quarter of the patients present with metastatic disease [5]. The Intergroup Rhabdomyosarcoma Study Group (IRSG) was formed in 1972 to systematically study the therapy and biology of children with rhabdomyosarcoma. The first study by the group was IRS-I (1972–1978) that evaluated the addition of doxorubicin to vincristine, dactinomycin, and cyclophosphamide (VAC) plus radiation [14]. The OS of those patients was 20%. The IRS-II study conducted 1978–1984 evaluated the use of repetitive cycles of VAC compared to alternating cycles of VAC and vincristine, doxorubicin, and cyclophosphamide [15]. Although the complete remission rate for those patients was 53% the OS was 26%. The IRS-III study, conducted between 1984–1991, examined the addition of cisplatin and etoposide to the previous regimens used in IRS-II, but failed to improve the survival of patients with group IV disease with 5-year OS of 27% [16]. Between 1991–1997, IRS-IV study evaluated the addition of ifosfamide and etoposide to VAC and the result also showed no significant improvement in the 3-year event free survival (25%) and 3-year OS (39%) of patients with group IV disease compared to prior studies [17]. Our findings in this study reflect the lack of improvement of outcome of those patients since 1980. The improvement observed in our study between the 1970s to 1980s likely reflects the introduction of IRS therapy in the 1970s.

Ewing sarcoma represents the second most common soft tissue sarcoma and the second most common primary bone sarcoma in children. Although the use of chemotherapy has significantly improved the outcome of patients with localized disease from an OS of <20% to 70–80%, it has not been as effective in patients with metastatic disease [3]. In the 1960s, patients were treated with single agent chemotherapy with dismal survival. In the 1970s, combination chemotherapy was used primarily consisting of VAC [18]. A large intergroup study was conducted from 1973 to 1978 that randomized patients to VAC with or without doxorubicin [19]. Patients with localized disease randomized to VAC plus doxorubicin had significantly improved survival compared to those receiving VAC alone. In a landmark study conducted between 1988–1992, patients were randomly assigned to receive 49 weeks of standard chemotherapy with doxorubicin, vincristine, cyclophosphamide, and dactinomycin or experimental therapy with these four drugs alternating with courses of ifosfamide and etoposide [20]. Although patients with localized disease had significantly improved survival with the addition of ifosfamide those with metastatic disease had no benefit with an OS of 22%. Although the survival of children with localized disease is 73% [21], the 10-year OS for children diagnosed after 1999 with metastatic disease in our study remains low at 30.6% with very limited improvement in survival since the 1980s and 1990s.

Osteosarcoma is the most common primary bone tumor in children and 15% to 20% of patients present with metastatic disease. The treatment prior to the 1970s consisted mainly of surgery with single agent chemotherapy [2]. Meyers et al reported on a study conducted from 1975 to 1984 using multi-agent neoadjuvant chemotherapy followed by surgery in children with metastatic disease but only 11% of the patients survived [22]. The survival improved in the 1990s to 29% with the addition of cisplatin to the chemotherapy regimen [10]. These results are consistent with our observation with survival improving from 10% in the 1980s to the 31% in the 1990s; however, no significant improvement has been made since the 1990s.

Although the improvement in patients with soft tissue sarcoma has been stagnant for the past two to three decades there has been consistent improvement in the survival in patients with metastatic neuroblastoma. We observed a significant increase in survival in every decade since the 1970s. This improvement is likely related to the intensification of multi-agent chemotherapy and radiation therapy for these patients [4]. In the 1990s, the introduction of autologous stem cell transplant (ASCT) and 13-cis-retinoic acid therapy improved the survival of high risk neuroblastoma patients [23]. In the past 10 years, survival was further improved with using immune therapy with Anti-GD2 therapy following ASCT [24]. Our data shows significant improvement in survival of patients who are older than 2 years of age at diagnosis as well as those who are less than 2 years. The survival of those who are less than 2 years of age is 71.5% while the survival of older patients, which generally have more biologically aggressive disease, remains low at 38.9%.

Wilms tumor is the most common renal tumor in children. Combination chemotherapy with vincristine and actinomycin D was used as early as the late 1950s and early 1960s [25]. The National Wilms Tumor Study (NTWS) group was established in 1968 to systematically study therapies for patients with Wilms tumor. The third NTWS (1979–1986) evaluated the addition of doxorubicin to the two drug regimen and resulted in an improved survival of 80% for patients with stage IV favorable histology disease [26]. The outcome for children with stage IV disease with diffuse anaplasia has remained poor with an OS of 33% in the most recent NTWS-5 study [27]. In our analysis the OS of children with stage IV disease has not improved since 1980s.

It is important to discuss the strengths and weaknesses of these data. The SEER database offers the unique ability assess outcome for large numbers of patients with these rare diagnoses who were treated throughout the United States over the last 40 years. Limitations to these data are that the SEER database does not provide details on the use of radiotherapy nor does it provide any information regarding the chemotherapy employed in the treatment of these children. Additionally, the SEER database provides limited biological information. While we are able to differentiate embryonal versus alveolar histology for many rhabdomyosarcoma patients, other biological information such as N-MYC amplification for neuroblastoma patients or anaplastic histology for Wilms tumor patients were either unavailable or available for very few patients. Another possible limitation to the data is the issue of stage migration in more modernly treated patients. However, we noticed no significant increase in the percentage of patients diagnosed with metastatic disease in any of the tumor types over the decades in this study. Our study is a comprehensive and provocative analysis of the outcome of children with advanced solid tumors over four decades.

The improvement in outcome for childhood cancer patients is a testament to the collaborative approach in pediatric oncology which extends nationally and internationally. However, with the exception of neuroblastoma, little improvement has been made in the last 20–30 years for children with metastatic solid tumors. These results reinforce the importance of continued collaborative efforts evaluating targeted therapy and further research to understand the underlying biology of these diseases.

### Key Message

Outcome for children with metastatic solid tumors has significantly improved since the 1970′s. However, with the exception of neuroblastoma, there has been little improvement in other solid tumors in the last 20–30 years.
